# Randomized controlled trial of the CMR immersive virtual reality (IVR) headset training compared to e-learning for operating room configuration of the CMR versius robot

**DOI:** 10.1007/s11701-024-01885-y

**Published:** 2024-03-30

**Authors:** Catherine L. Eley, Varun Palaniappan, Abbie Carter, Opeyemi Sogaolu, James Horwood, Michael Davies, Jared Torkington, James Ansell

**Affiliations:** 1 University Hospital of Wales, Heath Park, Cardiff, CF144XY United Kingdom; 2 Princess of Wales Hopsital, Coity Road, Bridgend, CF31 1RQ United Kingdom; 3 School of Medicine, Cardiff University, Heath Park, Cardiff, CF14 4XN United Kingdom

**Keywords:** Virtual Reality, Modular, Robotic, Surgery

## Abstract

Robotic surgery offers potential advantages over laparoscopic procedures, but the training for configuring robotic systems in the operating room remains underexplored. This study seeks to validate immersive virtual reality (IVR) headset training for setting up the CMR Versius in the operating room. This single-blinded randomized control trial randomised medical students with no prior robotic experience using an online randomiser. The intervention group received IVR headset training, and the control group, e-learning modules. Assessors were blinded to participant group. Primary endpoint was overall score (OS): Likert-scale 1–5: 1 reflecting independent performance, with increasing verbal prompts to a maximum score of 5, requiring physical assistance to complete the task. Secondary endpoints included task scores, time, inter-rater reliability, and concordance with participant confidence scores. Statistical analysis was performed using IBM SPSS Version 27. Of 23 participants analysed, 11 received IVR and 12 received e-learning. The median OS was lower in the IVR group than the e-learning group 53.5 vs 84.5 (*p* < 0.001). VR recipients performed tasks independently more frequently and required less physical assistance than e-learning participants (*p* < 0.001). There was no significant difference in time to completion (*p* = 0.880). Self-assessed confidence scores and assessor scores differed for e-learning participants (*p* = 0.008), though not IVR participants (*p* = 0.607). IVR learning is more effective than e-learning for preparing robot-naïve individuals in operating room set-up of the CMR Versius. It offers a feasible, realistic, and accessible option in resource-limited settings and changing dynamics of operating theatre teams. Ongoing deliberate practice, however, is still necessary for achieving optimal performance. ISCRTN Number 10064213.

## Introduction

Robotic surgery may offer several advantages over laparoscopic surgery, such as 3-dimensional vision, articulated wrist movement and improved ergonomics, but there is limited information on training for configuring robotic systems in the operating room [1], with new research mostly focussing on surgical technique. Immersive virtual reality (IVR) simulators enable cost-effective, portable, and realistic training in a resource constrained environment. In contrast to conventional virtual reality, IVR provides a continuous, scaled environment that can simulate the full extent of sensory stimuli perceived by users in the theatre environment [2]. IVR provides a completely interactive 3-dimensional (3D) simulation projected onto a head-mounted display (HMD), facilitating 360° visual immersion and instantaneous manipulation of virtual objects. This method of learning is gaining popularity as an alternative or adjunct to video learning for surgical training [3] and has been shown to facilitate transfer of skills to the theatre environment [4].

**Fig. 1:**
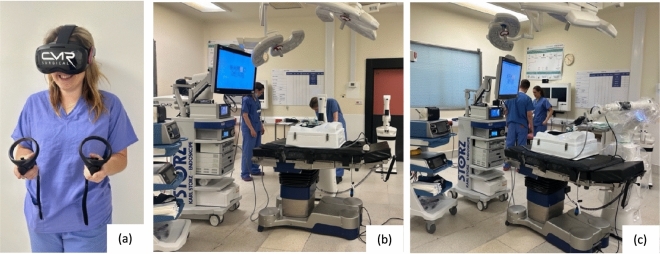
CMR Versius: Immersive Virtual Reality Headset (**a**) and robot modular bedside unit set-up (**b**, **c**)

The current surgical training pathway for the CMR Versius continues to be explored: currently surgeons complete 10 × E-learning modules; an optional 6 VR Headset modules; the Versius Trainer, with predefined benchmark metrics to achieve competence; and a 3-day cadaveric team training with the surgeon, first assist and two scrub staff. The VR Headset is a new addition (Fig. 1), but not yet compulsory component of the learning pathway. However, it is thought to be more engaging and intuitive than the didactic e-learning, allowing participants to complete ‘learn mode’ and subsequently ‘practice mode’ for each of the 6 modules to set up the robotic system in a simulated virtual reality operating room environment. Contrary to most other surgical simulation training tools, these modules focus predominantly on the set-up of the system, with an open console and modular design, allowing for freedom of port placement (Fig. 1), while surgical skill acquisition is demonstrated using the Versius Trainer (VT).

It is of high priority to establish a robust National Robotic-Assisted Training Programme with concentration on training and delivery. Sharing a small number of robots between surgical specialties; with a learning curve for mastering robotic procedures, additional armamentarium for training must be optimised. The face and content validity of these VR modules have yet to be established.

Aims: The aim of this randomized control trial is to compare current e-learning, with immersive VR for training personnel in the set-up of a modular robotic platform in theatre.

## Methods

### Study design

This is a single center, single-blinded randomized control trial of the CMR Immersive Virtual Reality (IVR) headset training compared to e-learning for teaching operating room set-up of the CMR Versius robot. The study was reviewed by the Cardiff University School of Medicine Research Ethics Committee and did not require ethical approval. The study was registered with ISRCTN (ISRCTN10064213).

### Participants

Medical students were invited to take part through Cardiff University Surgical Society. They were an accessible group of individuals where inclusion criteria necessitated no prior robotic experience, access to a laptop and internet connection at home, and visually able to use the IVR headset. Surgeons of all training grades have existing varied exposure to the use of the robotic platform in the workplace and therefore are not an entirely robot-naïve group of individuals. Moreover, medical students have previously served as a novice baseline in a study that validated the competency assessment of the CMR Versius trainer for surgical skills [5]. Exclusion criteria included those with prior education in setting up the CMR Versius robot, or those unable to use the IVR headset. Existing theatre staff were not included in the study due to the potential confounder of varying degrees of pre-existing robotic experience; with many having at least observed the robotic platform in operation; this deemed to possess a perceived advantage.

#### Randomisation

After verification of inclusion and exclusion criteria, upon receipt of the participation information leaflet and subsequent informed consent, participants were randomised by CE using an internet-based programme in a 1:1 ratio and issued with the appropriate details and equipment. Both assessors were blinded to the intervention and control group throughout.

Intervention group:

The intervention group received an IVR headset (Fig. 1), along with individualized Versius Connect portal log in details. Participants were asked to complete ‘learn mode’ and subsequently ‘practice mode’ for each of the 6 VR module. Modules covered moving and preparing the surgeon console for use, connecting the instrument and visualisation bedside units, draping to ensure sterility, instrument identification and attachment, port training and finally entering the robot into surgical mode. Participants were given a two-week period to complete the tasks prior to the operating room set-up assessment. Module information can be found in Appendix 1.

#### Control group

The control group received individualised Versius Connect portal log in details to access e-learning modules through any modern web browser. Modules relevant to operating room set-up were agreed upon by the protocol committee (found in Appendix 2) and participants were also given a two-week period to complete the tasks prior to the operating room set-up assessment. They covered the same objectives listed above.

#### Primary end points

The primary endpoint was the correct set-up of the modular robotic system in theatre, assessed using a modified, CMR validated Likert-scale assessment tool to devise an overall score of this performance by two independent assessors. This can be found in Appendix 3. A score of 1 equates to performing the task independently, 2–4 requires increasing degrees of verbal prompting ranging from passive questioning e.g., “What else might you look for?” to active suggestions e.g., “plug it into an un-interrupted power source” and 5 requiring physical assistance from the assessor to progress. Secondary endpoints were scores per task, time taken, inter-rater variability and concordance between score and participant confidence score. Ethical approval for this study was obtained from Cardiff University School of Medicine Research Ethics Committee (SMREC23/01).

### Statistical analysis

Based on similar published literature, to have a 90% chance of detecting a 20% decrease in overall score in the immersive VR group, which equates to a reduction of 1 Likert-score point per task, at the significance level of 0.05: 6 patients are required; 3 in each group [6].

IBM SPSS Version 27 was used to perform Mann–Whitney U and Chi squared tests to compare differences between intervention groups and Fleiss’ kappa to measure inter-rater reliability.

## Results

**Fig. 2:**
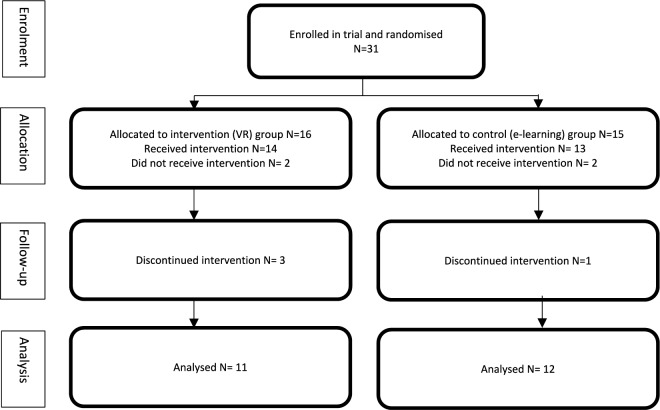
CONSORT diagram

Of 23 participants analyzed in the study, 11 received the intervention (VR headset) and 12 the control (e-learning): Fig. 2.

Two consistent raters assessed the participants throughout, with fair inter-relater reliability: *k* = 0.254 (95% CI: 0.214–0.294, *p* < 0.005). Agreement was higher when participants performed tasks independently without prompting (i.e., scored 1) (*k* = 0.419: 95% CI 0.354–0.485, *p* < 0.005).

**Fig. 3:**
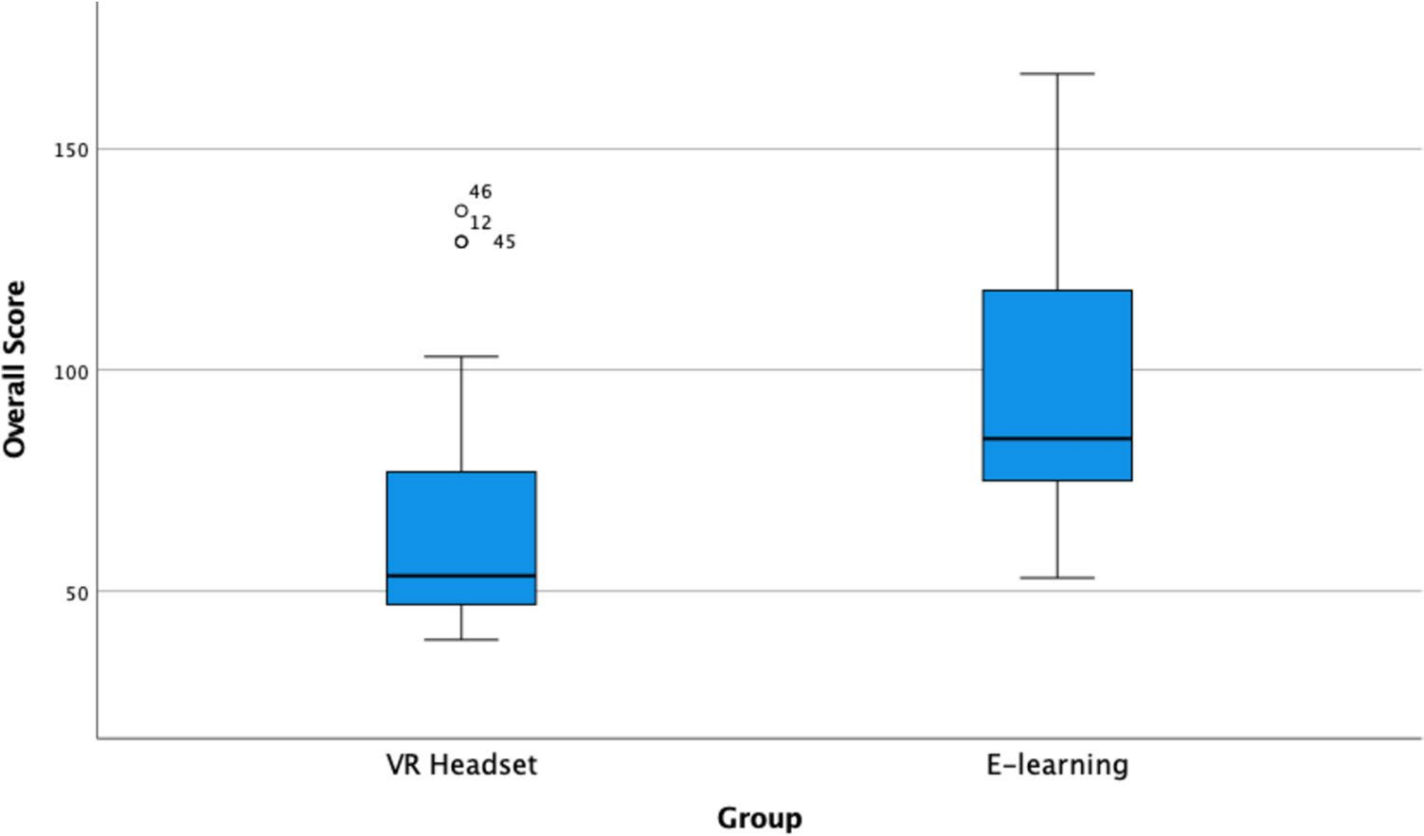
Boxplot showing the difference in overall score between VR group and e-learning

Overall median score was 77.7 (interquartile rage (IQR) 53 – 110.25) with a significant difference in overall score between VR group and e-learning group (median 53.5 (46.5–77.25), and 84.5 (73.5–118.5), respectively, *p* < 0.001) with lesser scores reflective of more independent performance (Fig. 3). Irrespective of learning method, the median score per task was 2 (1–3); VR intervention group; median = 2 (1–2) vs. e-learning control; median = 2 (2–3), *p* < 0.001.

**Fig. 4:**
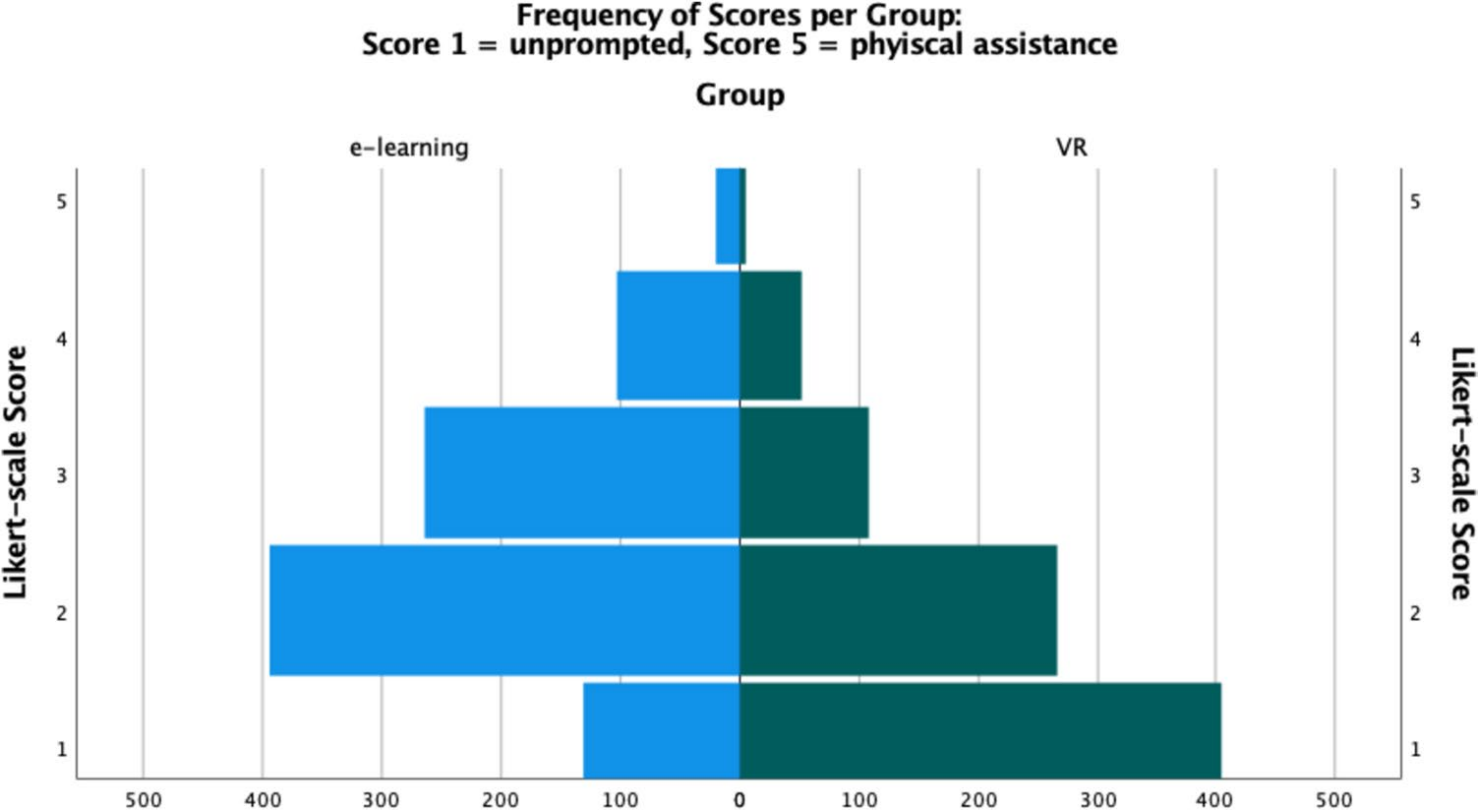
Frequency of Scores per intervention group: 1 — unprompted; 5 — physical assistance

VR recipients performed tasks independently more often than their e-learning counterparts (Score 1: VR 48.4% vs e-learning 14.4%, x^2^ 253.266, df 4, *V* = 0.381, *p* < 0.001), and required less frequent physical assistance (Score 5: VR 0.6% vs e-learning 2.2%, x^2^ 253.266, df 4, *V* = 0.381, *p* < 0.001) (Fig. 4). There remained a significant difference in performance between both VR- and e-learning- when compared to a ‘perfect score’, of unprompted throughout (*z* = − 18.584, *z* =− 24.738 respectively, *p* < 0.01). Time taken for completion of all tasks did not vary between groups (VR, median 22 min (20–26) vs e-learning 22.5 (19.25–27), *p* = 0.880). Results are summarized in Table 1.

**Table 1:** Univariable analysis of theatre set-up outcomes using VR vs. e-learning

	VR	e-learning	*p*-value
Overall score (Median (IQR))	53.5 (46.5–77.25)	84.5 (73.5–118.5)	< 0.001
Individual task performance (Median (IQR))	2 (1–2)	2 (2–3)	< 0.001
Independent task success: Score = 1 (*n* (%))	405 (48.4%)	131 (14.4%)	< 0.001
Physical assistance needed: Score = 5 (*n* (%))	5 (0.6%)	20 (2.2%)	< 0.001
Time in minutes (median (IQR)	22 (20–26)	22.5 (19.25–27)	0.880

**Fig. 5:**
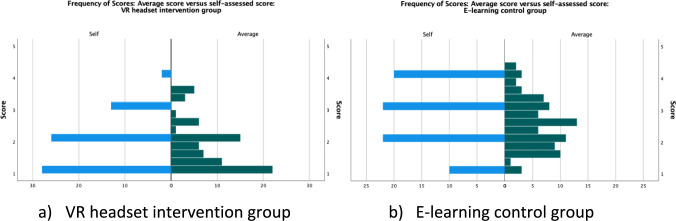
Frequency of Average scores versus self-assessed score per intervention group: **a** VR headset, **b** e-learning (1 —unprompted; 5 —physical assistance)

There was no statistically significant variation in average task-score and self-assessed confidence score in the VR group (median average score 1.67 vs self-assessed score 2, *p* = 0.607), as opposed to a discernible difference in scores in the control e-learning group (median average score 2.5 vs self-score 3, *p* = 0.008) (Fig. 5).

## Discussion

Robotic Surgery set-up requires sufficient user-education and is currently an unstudied area in existing research. This is the first study to look at educational materials to teach robot-naïve individuals to configure the modular CMR robot in a theatre environment (Fig. 1). The principal finding is immersive VR learning can prepare robot-naïve individuals with little-to-no prompting, better than e-learning materials. It should be noted, however, that both modalities educate learners to the level requiring minimal prompting. Robotic surgery set-up requires additional considerations for which surgical and theatre staff require additional training. Port placement for pelvic surgical procedures may differ from their conventional laparoscopic arrangement due to the need to maintain a clear arc of operation around the pivot point for which individual robotic arms have been trained. Theatre staff and surgeons must be well versed in set-up to achieve this efficiently and safely. Considering elements such as robotic console and arm positioning, optimal port placement and cabling, orientation, start-up, and shutdown, as well as storage and troubleshooting can all significantly impact technical performance, and safety in the operating theatre. This skill set differs from conventional laparoscopy and otherwise highly experienced staff may require extensive training to work with robotic systems. Moreover, modular systems with many individual moving parts increase configuration workloads, with set-up requirements manufacturer and procedure-specific, increasing the skill sets required further.

Surgeon’s acquisition of technical skill is monitored via stringent training programmes which incorporate simulation and proctoring. Limited capacity, however, exists for nursing staff to access team-training exercises, and in addition to regular staff turnover, these restrict wider theatre nurse education in robotic set-up [7]. These evolving technical challenges added to nursing and practitioner roles compound the importance of educating a larger pool of less experienced team members [8]. This situation has put increased pressure on education and training outside of the operating room to ensure all team members are adequately prepared to handle the diverse demands of their roles.

Previous research has focussed on robotic surgical skill acquisition and transfer [9–17] with the expansion of available robotic platforms. Aside from CMR Versius, other VR simulation platforms include Robotic Surgery System (Simulated Surgical Systems, United States), the dV-Trainer (Mimic, United States), the da Vinci Skills Simulator (Intuitive Surgical, United States), and RobotiX Mentor (3D Systems, United States) [18]. None of these have explored the use of wearable head-mounted VR technology and existing research neglects operative room set-up. Pan-theatre, pan-specialty use of the robot makes their resource for training this skill set in-theatre an unrealistic sustainable option; it’s necessary to overcome these limited opportunities for hands-on training and scarcity of robotic equipment outside of the operating theatre. Adjuncts such as VR headsets can provide realistic and interactive learning scenarios in a virtual environment that closely mimics real-world situations, allowing users to develop, practice and refine their skills, without the need for expensive equipment or dedicated physical spaces, accessible and feasible for implementation in resource-limited settings [9–16].

The strength of this study shows that with a short 2-week period of either learning modality, skill transfer can occur in complete novices with a small number of verbal prompts required.

The limitations are two-fold: this study used a relatively small sample size, however, with a paucity of available evidence, the intent of this study was to provide some pilot data and test the feasibility of such study of educational materials for theatre configuration. Secondly, the application of the modified score sheet, using a Likert-scale of 1–5 could have been more descriptive to make absolute conclusions; this is reflected further by a higher inter-rater reliability for individuals requiring no prompts, as this is less subjective than amount of verbal prompting required, scoring between points 2 and 4.

VR trained individuals appear better equipped to self-assess their performance than the e-learning group. This may attribute to the immersive and realistic training environment that VR provides, with active engagement and real-time feedback, allowing for adjustment and corrections; perhaps not offered to the same degree using e-learning platforms. Moreover, repetition using VR increases exposure, can encourage pattern recognition and correction, promoting self-awareness, self-reflection and as a result improved self-assessment. Despite this, as one may expect, ongoing deliberate practice over time is still required to work towards a ‘perfect score’ [19, 20].

## Conclusion

This study is the first to concentrate on operative room configuration in an evolving robotic surgical climate. Immersive VR learning was found to be more effective than e-learning for preparing robot-naïve individuals in operating room set-up of the CMR Versius. With a growing pool of less experienced team members, variable staff retention, and changing dynamics of operating theatre teams this study is important in expanding the armamentarium of educational resource development and training. IVR can provide a realistic, interactive learning environment, offering a feasible and accessible option in resource-limited settings, whereby individuals are better equipped to self-direct their learning and self-assess performance. However, IVR does not replace benefits of ongoing deliberate practice over time and is still necessary for achieving optimal performance.

## Data Availability

Data is available on request.

## Appendix 1:

Module 1: Surgeon console.

Module 2: Connecting the system.

Module 3: Bedside units.

Module 4: Draping.

Module 5: Versius Principles.

Module 6: Safety features.

## Appendix 2:

Video Module 1: System set-up.

Video Module 2: Versius concepts.

Module 1: introduction to Versius surgical system.

Module 2: The Versius surgeon console.

Module 3: The Versius bedside units.

Module 4: Versius bedside unit set-up.

Module 5: Connecting the system.

Module 6: Draping the bedside units.

Module 7: Introduction to arm mode icons.

Module 8: Using Versius arm modes.

*These modules were excluded as they do not relate to the operating room set-up and these components were not assessed.

## Appendix 3: Likert score marking sheet. Copyright 2022–2023 by CMR Surgical Limited.

**Table:**

Surgeon console and bedside unit set-up						
Task	Steps/actions	1	2	3	4	5
Moved the surgeon console safely	Hand controllers are docked while the system was moved and there were no trailing cables					
	The surgeon console was in storage position while moved					
Surgeon console set-up completed	The console power cable is connected to an uninterrupted power supply (UPS)					
	The network cable is connected					
	The white auxiliary cable is connected to the console and auxiliary screen					
	The arm rests have been extended on the surgeon console					
	The surgeon has a chair with a stable base					
System is cabled safely and accurately	IBSU and VBSU recognized in storage and correct BSUs have been selected for procedure					
	No cables are running under the patient bed					
	No cables are blocking doors or exits					
	The cables were plugged in confidently (no alarms were set off during cabling)					
	The purple video feed cable is connected from the surgeon console to the VBSU					
Arms are draped and sterile	During draping, the arms were moved safely—without putting hands in hand traps and using black grip band for large movements					
	Arm positioned for draping					
	Lock and unlock, yes and no sounds were recognised					
	IBSU drape caps are correctly fitted with no red marks showing and the locking ring is fully pulled back					
	IBSU drape cap insert is correctly fitted with no red dots showing					
	VBSU drape cap is correctly fitted and secured					
	The tethers are secured in the correct places					
	All the coloured tapes are removed					
	BSU arms are completely sterile					
	BSU have been locked in low profile position					
Camera is draped and sterile	The endoscope is correctly connected to the camera, clicked firmly into place					
	The camera cable was not held or damaged while draping					
	The blue tapes were secured in the correct position					
	Camera is sterile					
	The correct endoscope angle has been selected on the HUD menu					
Start-up Checklist	The “Before Patient Enters the Operating Theatre” checklist is verbalised and actioned					

**Table:**

Task	Steps/actions	1	2	3	4	5
Position the bedside units	Bedside units are positioned parallel to the bed					
	The brake was activated while checking for obstructions and is facing outwards for easy access					
	The shoulder of the bedside unit is positioned in line with the top of the port					
Attach instrument and connect electrosurgery cable (if applicable)	Attached instrument using 3-point check					
	All electrosurgery cables are connected					
	Electrosurgery settings have been checked using the Instruments and Accessories Manual					
Port Training	All the BSU arm joints are bent, and arm is positioned for port training					
	While positioning the arm the hand traps were avoided, black grip band used for large movements					
	While the BSU arm was unlocked it was always supported					
	When the instrument was placed in the port, the port was supported, and the user did not hold the instrument shaft					
	The instrument was in the correct position for port training—at the trocar sleeve tip and facing the surgical site					
	The user was watching the port site during port training and made the correct movement, circular for IBSU and U-shape for VBSU					
	The user recognised successful port training when the port training icon becomes static					
Navigate to Surgical mode	User transitions to instrument adjust using the V-Wrist button					
	User is able to guide instrument into the patient cavity under vision					
	User moves more than 2 cm before activating surgical mode and communicates control is passed to the surgeon					
Start-up Checklist	When all BSU’s are in surgical mode the “Before Surgery” checklist is verbalised and actioned with the team					

*For this exercise, to limit resource waste, the camera was pre-draped, sterile and positioned, and no electrosurgery was used.
